# Effect of Radiation Therapy on Composition of Lymphocyte Populations in Patients with Primary Breast Cancer

**DOI:** 10.3390/jpm13091399

**Published:** 2023-09-19

**Authors:** Irina Kobzeva, Tatiana Astrelina, Yuliya Suchkova, Tatyana Malivanova, Daria Usupzhanova, Vitaliy Brunchukov, Anna Rastorgueva, Victoria Nikitina, Ekaterina Lubaeva, Marina Sukhova, Alexey Kirilchev, Tatyana Butkova, Alexander Izotov, Kristina Malsagova, Alexander Samoilov, Vasiliy Pustovoyt

**Affiliations:** 1 State Research Center—Burnasyan Federal Medical Biophysical Center of Federal Medical Biological Agency, 141701 Moscow, Russia; irina-kobzeva@yandex.ru (I.K.); t_astrelina@mail.ru (T.A.); yuls11349@yandex.ru (Y.S.); tmalivanova@yandex.ru (T.M.); usupzhanova94@mail.ru (D.U.); brunya2008@yandex.ru (V.B.); rastorgueva.ann@gmail.com (A.R.); nikitinava@yandex.ru (V.N.); katrin.radiolog@yandex.ru (E.L.); mysuchova@fmbcfmba.ru (M.S.); radiationtherapistt@gmail.com (A.K.); asamoilov@fmbcfmba.ru (A.S.); vipust@yandex.ru (V.P.); 2 Institute of Biomedical Chemistry, Biobanking Group, 109028 Moscow, Russia; t.butkova@gmail.com (T.B.); izotov.alexander@gmail.com (A.I.)

**Keywords:** lymphopenia, breast cancer, radiotherapy, lymphocytes

## Abstract

Background: Radiation therapy (RT) is an important step in the treatment of primary breast cancer as it is one of the leading contributors to cancer incidence among women. Most patients with this disease acquire radiation-induced lymphopenia in the early post-radiation period; however, little is known about the effect of RT on the composition of lymphocyte populations in such patients. This study was aimed at investigating the effect of adjuvant remote RT—performed in the classical mode for patients with primary breast cancer—on the main components of cell-mediated immunity (major lymphocyte populations), including those in patients receiving chemotherapy. Methods: Between 2020 and 2022, 96 patients with stage I–III breast cancer were included in this study. All patients in the final stage of complex treatment received RT via a 3D conformal technique (3DCRT). The clinical target volume of this RT included the breast or chest wall and locoregional lymphatics. Flow cytometry was used to assess the levels and phenotypes of circulating lymphocytes before and after RT (no more than 7 days before and after RT). The evaluation of the impact of polychemotherapy (PCT) was conducted to determine whether it was a risk factor for the onset of radio-induced lymphopenia (RIL) in the context of RT. Results: When assessing the immune status in the general group of patients (n = 96), before the start of adjuvant external beam radiotherapy (EBRT), the average number of lymphocytes was 1.68 ± 0.064 × 10^9^/L; after the course of adjuvant EBRT, it decreased to 1.01 ± 0.044 × 10^9^/L (*p* < 0.001). When assessing the absolute indicators of cellular immunity in the general group of patients with BC after a course of adjuvant EBRT, significant dynamics were revealed by the changes in all cell populations of lymphocytes (paired *t*-test, *p* < 0.05). Conclusion: The adaptive immune system in breast cancer patients changed in the early post-radiation period. The absolute levels of B-, T- and natural killer cells significantly reduced after RT regardless of whether the patients previously underwent chemotherapy courses. RT for patients with primary breast cancer should be considered in clinical management because it significantly alters lymphocyte levels and should be considered when assessing antitumor immunity, as significant changes in T-cell immunity have been observed. In addition, the identified changes are critical if specific targeted therapy or immunotherapy is needed.

## 1. Introduction

Currently, the treatment of oncological diseases is inextricably linked to the use of radiation therapy (RT). Such therapy is an integral component of complex antitumor treatment for breast cancer (BC), which is a leading cause of oncological morbidity among women [1]. In most cases, RT is used as the final or adjuvant stage in the treatment of primary BC, after surgery and drugs, to improve local control of tumorigenesis [2].

In the same time, despite all the measures taken to protect normal tissues from exposure to ionizing radiation, there is still a risk of developing complications during RT, including complications caused by the destruction of radiosensitive cells of the immune system (IS) circulating through the radiation portal. Many researchers have reported the development of radiation-induced lymphopenia (RIL) as one of the main side effects of remote RT for oncological diseases, including BC [3,4,5,6,7,8].

IS dysfunction not only plays an important role in the pathogenesis of the initial response of human tissues to irradiation, but it is also an unfavorable prognostic factor for the overall survival of patients with solid tumors, which may result from altered antitumor immunity [7,8,9,10].

According to a number of studies, damage to IS cells induced by irradiation for BC leads to the immunosuppressive state and reduces the effectiveness of further antitumor therapy [6,11,12].

Several researchers consider RIL a prognostically unfavorable factor for overall survival among patients from certain clinical groups of BC [13,14,15,16,17]. In addition, a number of studies have demonstrated a direct correlation between the level of lymphocytes in peripheral blood and the response rate to therapy with immune checkpoint inhibitors for patients with BC [3,6].

The degree of damage to IS cells directly depends on both the physical factors of ionizing radiation (the nature of exposure, power levels and doses, and relative biological effectiveness of radiation) and the initial general condition of the patient and their IS, the presence of previous drugs, and/or the administration of immunotherapy [3,5,14,18].

Given that approximately 30–40% of patients with primary BC require systemic polychemotherapy (PCT) as one of the stages of antitumor treatment, its effect on the degree of RIL has recently become an international topic of discussion [18,19]. The authors of these studies identified PCT as a risk factor for the development of RIL, which should be considered when planning programs and the timing of RT.

Despite studies being devoted to the development of RIL and its impact on the prognosis of BC, there are many unresolved issues. The literature sources practically do not consider the effect of local RT for primary BC on the composition of lymphocyte populations, which play a certain role in the implementation of the antitumor immune response (altered lymphocyte population composition may alter the immune system’s antitumor response, particularly due to the dysfunction of cellular links, and it also affects further anti-tumor therapies). Such studies were conducted only at the end of the 80s; they have not been presented in the literature since [20,21].

The aim of this study was to evaluate the effect of adjuvant external beam radiotherapy (EBRT) on the main indicators of cellular immunity (lymphocyte populations and their composition) in patients with primary BC.

## 2. Materials and Methods

### 2.1. Patients

From 2020 to 2022, 96 patients with a histologically verified diagnosis of BC, stages I–III, and who received adjuvant EBRT via a 3D conformal technique (3DCRT) were enrolled in this study. The exclusion criteria were concurrent chemoradiotherapy, noninvasive BC, stage IV of the oncological process, the presence of malignant neoplasms of other localization and immunological diseases, and refusal to participate in the protocol.

The mean age of the patients was 57.4 ± 13.31 (range: 28–87) years. Stage I, II, and III disease was detected in 30.2%, 46.9%, and 22.9% of the patients, respectively.

According to the molecular subtype of the tumor, the patients were classified as follows: 24% were diagnosed with luminal A breast cancer, 51.0% with luminal B breast cancer (HER2−), 6.3% with luminal B breast cancer (HER2+), 8.3% with HER2, and 7.3% in triple-negative breast cancer. By histological type, nonspecific BC, lobular cancer, and other types of cancer were detected in 79.2%, 14.6%, and 6.3% of patients, respectively. According to the degree of malignancy of the tumor, the classification was as follows: G1—11.2%, G2—64.0%, G3—24.7%, and CIS—3.1%.

All patients underwent surgery for BC during the first stage of anti-tumor treatment. Only surgical intervention for BC was performed before adjuvant EBRT for 33 patients (34.4%); an additional stage of antitumor treatment, systemic PCT with taxanes, and anthracyclines and doxorubicin was performed for 63 patients (65.6%).

### 2.2. Assessment of Indicators of Cellular Immunity

To assess the dynamics of the IS, all patients underwent venous blood sampling before and after adjuvant EBRT 27 ± 3 days after the completion of surgical/chemotherapeutic treatment. Cellular immunity parameters were analyzed on the day of blood sampling by determining the absolute number and percentage of lymphocyte subpopulations with a flow cytometer (BD FACS Canto II, Becton Dickinson and Company, Franklin Lakes, NJ, USA), using a single-platform method with a commercial kit of monoclonal antibodies (Multitest IMK kit with BD Trucount, Becton Dickinson tubes) according to the manufacturer’s instructions. The results were evaluated using BD FACS Diva 6.0 software. 

Normal indicators accepted in our laboratory of cellular immunity are shown in Table 1.

The obtained data were statistically processed using the Statistica 6.0 software. The data between groups were compared using the two-tailed Fisher’s exact test. The mean values of the immune status parameters were expressed as means ± SEM and compared using the unpaired Student’s *t*-test; the paired *t*-test was used to analyze the dynamics of the parameters. For all criteria, the differences were considered statistically significant at *p* < 0.05.

## 3. Results

### Assessment of the Immune Status before the Start of Adjuvant External Beam Radiotherapy

At the first stage of the work, we assessed the effect of RT on the main indicators of cellular immunity in all patients in this sample.

When assessing the immune status in the general group of patients (n = 96), before the start of adjuvant EBRT, the average number of lymphocytes was 1.68 ± 0.064 × 10^9^/L; after the course of adjuvant EBRT, it decreased to 1.01 ± 0.044 × 10^9^/L (*p* < 0.001). Prior to the onset of adjuvant EBRT, 19.4% of patients had moderate lymphopenia, 77.2% had lymphocyte levels within the normal range, and 3.4% exhibited the above-normal values. After adjuvant EBRT, lymphopenia was observed in 74% of the cases.

**Figure 1:**
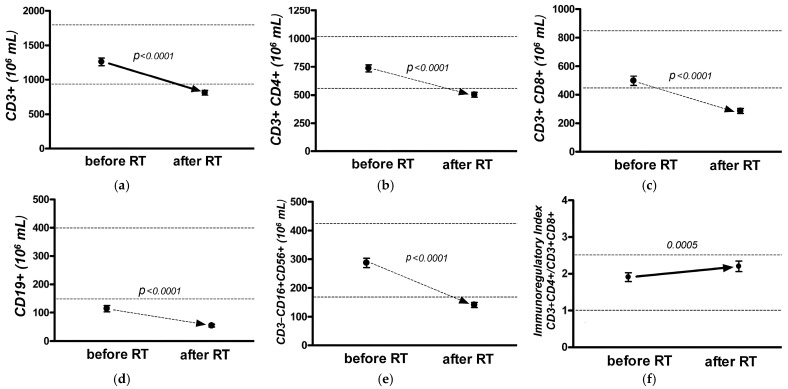
Changes of cellular immunity in the group of patients with breast cancer (BC): The schedules show alterations of T-lymphocyte (CD3+) (**a**), T-helper (CD3+ CD4+) cells (**b**), T-cytotoxic (CD3+ CD8+) cells (**c**), B-lymphocytes (CD19+) (**d**), natural killer (NK) cells (CD3− CD16+ CD56+) (**e**), and immunoregulatory index (CD4+/CD8+ ratio) (**f**) before and post RT.

When assessing the absolute indicators of cellular immunity in the general group of patients with BC after a course of adjuvant EBRT, significant dynamics were revealed by changes in all cell populations of lymphocytes (paired *t*-test, *p* < 0.05) (Figure 1a–f).

Thus, by the end of adjuvant EBRT, a significant decrease in the absolute number of B-lymphocytes (CD19+) and a decrease in the absolute number of natural killer (NK) cells (CD3− CD16+ CD56+) per unit of blood volume was recognized (Figure 1d,e). 

It is worth noting a deficiency in B-cell immunity in the general group before the start of adjuvant EBRT (Figure 1d). The number of T-lymphocytes (CD3+) after adjuvant EBRT significantly decreased (Figure 1a) due to a decrease in the number of both T-helper (CD3+ CD4+) cells (Figure 1b) and T-cytotoxic (CD3+ CD8+) cells (Figure 1c). The immunoregulatory index (CD4+/CD8+ ratio) increased after adjuvant EBRT but remained within the normal values (Figure 1e).

**Figure 2:**
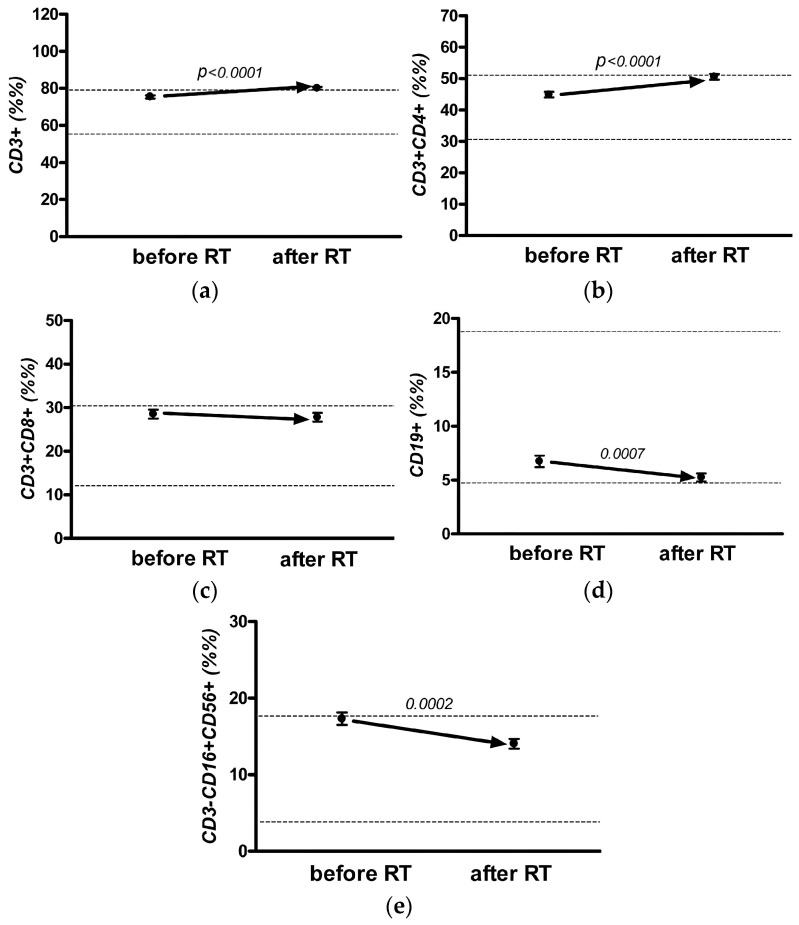
Dynamics of relative indicators of cellular immunity in the general group of patients with breast cancer (BC): T-lymphocyte (CD3+) (**a**), T-helper (CD3+ CD4+) cells (**b**), T-cytotoxic (CD3+ CD8+) cells (**c**), B-lymphocytes (CD19+) (**d**), and natural killer (NK) cells (CD3− CD16+ CD56+) (**e**) before and post RT.

Significant differences were observed in the relative values for all cell populations of lymphocytes (Figure 2a–d) (paired *t*-test, *p* < 0.05), except for T-cytotoxic (CD3+ CD8+) cells (Figure 2c). The percentage of T cells in the total population of lymphocytes after adjuvant EBRT reached the upper limit of the reference values (80.06 ± 0.71%), mainly due to the increase in the proportion of the T-helper (CD3+ CD4+) subpopulation (Figure 2b), whose indicators also reached the upper limit of the reference values (50.52 ± 0.94%).

The relative number of T-cytotoxic cells (CD3+ CD8+) did not change during RT (Figure 2c). The relative content of B-lymphocytes (CD19+) at the end of adjuvant EBRT significantly decreased but remained within the reference values (Figure 2d). The relative indices of NK cells (CD3− CD16+ CD56+) remained within the normal range during adjuvant EBRT (Figure 2e).

Thus, in the first stage of the study, when assessing the main indicators of cellular immunity, lymphopenia accompanied by a change in the composition of lymphocyte populations was detected in the general group of patients with BC after adjuvant EBRT.

The second stage of the work evaluated the assessment of the influence of systemic polychemotherapy (PCT) on indicators of cellular immunity after adjuvant external beam radiotherapy.

To assess the effect of previous systemic PCT on the parameters of cellular immunity after adjuvant EBRT, all patients were divided into two groups depending on the type of treatment performed (patients with PCT and those without).

In both study groups, the average lymphocyte count before the start of adjuvant EBRT was within the normal range (1.69 ± 0.087 and 1.67 ± 0.088 × 10^9^/L in the groups with and without PCT, respectively).

Prior to the start of adjuvant EBRT, in the group of patients without previous PCT, already 22.2% exhibited a decrease in lymphocyte counts below the normal range, 75% had normal lymphocyte counts, and 2.8% had lymphocyte counts above 3.0 × 10^9^/L. In the group with PCT, lymphopenia was detected in 14% of the patients; the lymphocyte count was within the normal range in 82% of the patients and above the normal range in 4% (Table 2).

In the early post-radiation period, both groups showed a decrease in lymphocytes (0.99 ± 0.05 and 1.03 ± 0.09 × 10^9^/L in the groups with and without PCT, respectively). This decrease in indicators was statistically significant for both groups (paired *t*-test, *p* < 0.0001).

An assessment of the distribution of lymphocytes within the groups yielded the following data: in the group without PCT, 75% of patients exhibited a decrease in lymphocyte count below 1.2 × 10^9^/L; the indicators were within the normal range for the other 25%. In the group with PCT, the situation was almost the same—77% of patients had lymphopenia and 23% exhibited lymphocyte levels within the reference values. We found that PCT did not have any effect on the total number of lymphocytes after adjuvant EBRT (*p* > 0.05), whereas adjuvant EBRT led to a significant decrease below the normal values (paired *t*-test, *p* < 0.001).

**Figure 3:**
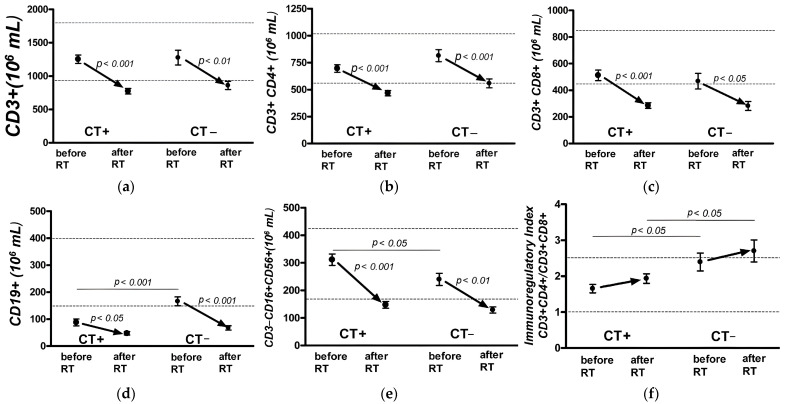
Dynamics of absolute indicators of cellular immunity in patients with breast cancer with and without polychemotherapy: T-lymphocyte (CD3+) (**a**), T-helper (CD3+ CD4+) cells (**b**), T-cytotoxic (CD3+ CD8+) cells (**c**), B-lymphocytes (CD19+) (**d**), natural killer (NK) cells (CD3− CD16+ CD56+) (**e**), and immunoregulatory index (CD4+/CD8+ ratio) (**f**) before and post RT.

An analysis of the absolute number of lymphocyte populations in patients in both groups, with and without PCT (Figure 3a–f), indicated a significant negative trend for all the studied parameters (paired *t*-test, *p* < 0.05), with the mean values exceeding the lower border of the norm.

The groups did not differ in terms of the content of T cells (Figure 3a), *p* > 0.05. In both groups, the absolute counts of T cells (CD3+) before the start of adjuvant EBRT were within the normal values. However, by the end of adjuvant EBRT, all patients showed a decrease beyond the normal values. This was due to a decrease in the populations of T-helper (CD3+ CD4+) cells in the group of patients with PCT and at the lower limit of the norm in the group without PCT (Figure 3b) and a pronounced decrease in the number of T-cytotoxic (CD3+ CD8+) cells in both groups (Figure 3c).

No statistically significant dynamics of changes in the level of immunoregulatory index indicators in either group were observed (Figure 3f), whereas the immunoregulatory index indicator in the group of patients without PCT was significantly higher than that in the group of patients who underwent PCT and after adjuvant has been shown.

A statistically significant suppression of the absolute number of B-lymphocytes (CD19+) before adjuvant EBRT was observed in patients in the group with previous PCT (Figure 3d). The level of B-lymphocytes in this group was below the reference value, whereas it was at the lower limit in the group without PCT (unpaired *t*-test, *p* < 0.001). After adjuvant EBRT, there was a decrease in this indicator beyond the lower limit of the norm to the comparable, statistically insignificant values in both groups.

Similarly, the level of NK cells (CD3− CD16+ CD56+) (Figure 3e) in patients in both groups before the start of adjuvant EBRT was within the normal values; however, there were statistically significant differences between the groups after the therapy. After adjuvant EBRT, the number of cells decreased below the normal values in both groups.

**Figure 4:**
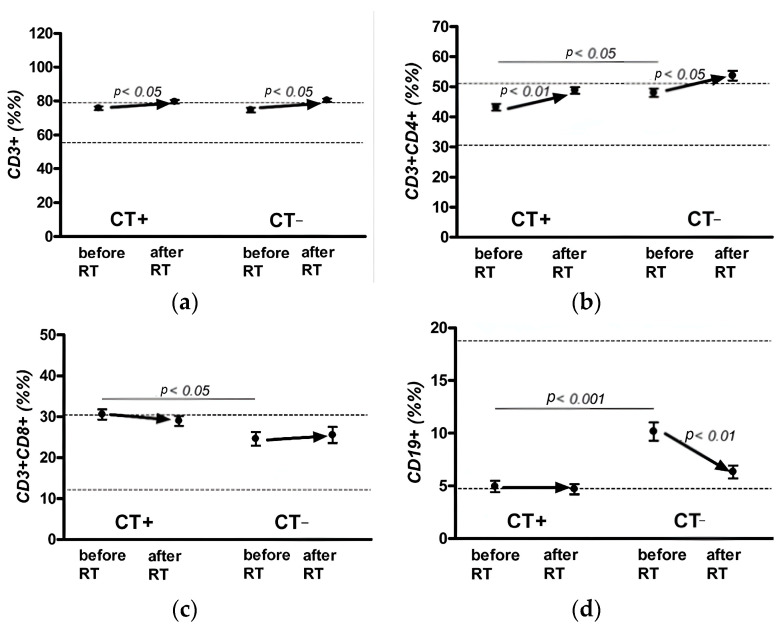

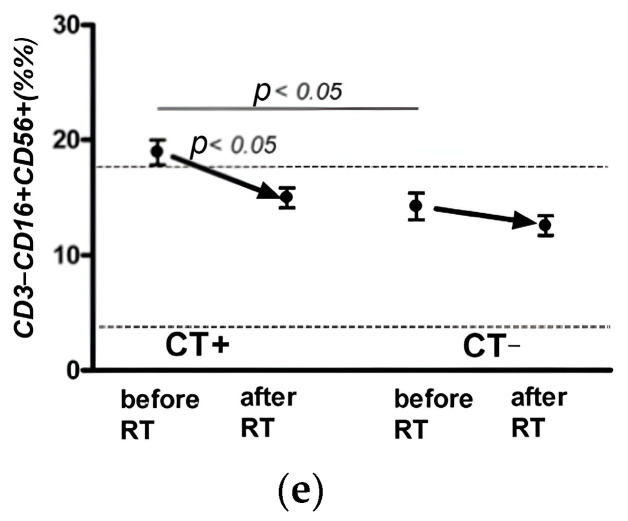
Dynamics of relative indicators of cellular immunity in patients with breast cancer with and without polychemotherapy: T-lymphocyte (CD3+) (**a**), T-helper (CD3+ CD4+) cells (**b**), T-cytotoxic (CD3+ CD8+) cells (**c**), B-lymphocytes (CD 19+) (**d**), natural killer (NK) cells (CD3− CD16+ CD56+) (**e**) before and post RT.

When analyzing the relative indicators of the immune status in patients from the two groups (Figure 4a–e), a significant dynamic was observed (paired *t*-test, *p* < 0.05) for almost all indicators, except for the content of T-cytotoxic cells (CD3+ CD8+) (Figure 4c) and NK cells (CD3− CD16+ CD56+) (Figure 4e) in the group of patients without PCT, and that of B-lymphocytes (CD19+) in the group of patients with PCT.

No differences were found between the two groups of the relative indices of T cells (CD3+) (Figure 4a). An assessment of the relative content of T-lymphocyte populations indicated that the level of T-cytotoxic (CD3+ CD8+) lymphocytes (Figure 4c) in both groups during adjuvant EBRT did not exhibit significant shifts, remaining practically within the normal values (at the upper limit of normal for the group with PCT). The relative indices of T-helper (CD3+ CD4+) cells (Figure 4b) also differed significantly between the two groups before adjuvant EBRT (*p* < 0.05).

The percentage of B-lymphocytes (CD19+) (Figure 4d) in patients from the group without PCT after adjuvant EBRT decreased from the normal values to the level of the lower limit of the normal values (paired *t*-test, *p* < 0.01). In the group of patients with PCT, the percentage of cells did not change during RT and was at the lower level of the normal values (Figure 4d).

Furthermore, the percentage of B-lymphocytes before adjuvant EBRT was significantly different between the two groups (*p* < 0.001). The level of NK cells (CD3− CD16+ CD56+) in the group (Figure 4e) of patients with PCT before adjuvant EBRT was slightly higher than the normal values and decreased to normal after adjuvant EBRT (paired *t*-test, *p* < 0.05); before adjuvant EBRT, the level of NK cells in this group significantly differed from that in the group without PCT (Figure 4e).

## 4. Discussion

Adjuvant EBRT is an important step in the treatment of primary BC. Despite the routine nature of this treatment method, information on its effect on IS cells is limited to a few publications [3,18,19,20,22]. In most cases, in the early post-radiation period, RIL develops to one degree or another due to the high radiosensitivity of immunocompetent cells passing through the radiation field. The impact of ionizing radiation on these cells causes defects in various parts of the IS.

Currently, RIL is considered an unfavorable prognostic factor in certain clinical variants of BC and should be taken into account when prescribing modern targeted drugs [23,24] and when using immunotherapeutic agents. Obtaining and expanding knowledge about the state of the IS and changes in the composition of lymphocyte populations in patients both before and after RT is an urgent task in modern oncology.

In this study, we evaluated the effect of adjuvant EBRT via a 3D conformal technique (3DCRT) on the main indicators of cellular immunity in 96 patients with primary BC, both after systemic PCT and without it.

After adjuvant EBRT, 77.2% of patients exhibited a decrease in lymphocyte counts. These results are consistent with the previously obtained data [3,22]. However, the data on the changes in the population composition of lymphocytes are not presented in the literature available to us. Thus, the obtained data on the effect of ADLT on the population composition of lymphocytes will be presented for the first time.

The compositions of lymphocyte populations after adjuvant EBRT revealed significant changes in the parameters of all cell populations of lymphocytes—B-lymphocyte, T-lymphocyte, and NK cells (paired *t*-test, *p* < 0.05). Thus, by the end of adjuvant EBRT, a decrease in the absolute number of B-lymphocytes (CD19+) per unit volume of blood, NK cells, and CD3+ lymphocytes were recorded due to a decrease in the number of both T-helper (CD3+ CD4+) cells and T-cytotoxic (CD3+ CD8+) lymphocytes.

The immunoregulatory index after adjuvant EBRT significantly increased. It should be noted that significant changes in the relative content of lymphocyte populations were not observed; this must be considered when interpreting the immunogram results.

An evaluation of the effect of previous systemic PCT on the main indicators of cellular immunity at this stage of the work did not reveal its effect on the total number of lymphocytes after RT, as in other works [4] (non-partner *t*-test, *p* > 0.05); however, adjuvant EBRT led to a significant decrease below the reference values of these indicators (paired *t*-test, *p* < 0.001).

When studying the composition of lymphocyte populations in both groups of patients, a significant decrease in the absolute counts of all the studied cell populations of lymphocytes was observed after a course of RT (paired *t*-test, *p* < 0.01).

A significant statistical difference in the immunoregulatory index between the groups of patients was found both at the beginning and end of adjuvant EBRT; however, there was no significant difference in the change in this indicator within the groups, although it had a tendency to increase after therapy. This reflects the general state of T cell immunity in patients after adjuvant EBRT.

There was also a significant decrease in the absolute number of B-lymphocytes and NK cells after EBRT for all groups of patients, regardless of PCT.

An analysis of the dynamics of the relative parameters of the immune status in patients from the two groups indicated no significant deviations, whereas a significant dynamic was observed (paired *t*-test, *p* < 0.05) for almost all indicators, except for the content of T-cytotoxic and NK cells in the group of patients without PCT, and that of B-lymphocytes in the group of patients with PCT.

## 5. Conclusions

This study showed that the adaptive IS in patients with primary invasive BC changes during the early post-radiation period. Patients develop RIL, which is accompanied by a significant decrease in almost all the studied absolute parameters of the immunogram and does not depend on the previous courses of PCT. Taking into account the timing of the studies to assess the main indicators of cellular immunity, we also exclude the impact of surgical intervention, since according to the previous studies, changes are observed at an earlier time after surgery [25].

It should be noted that we did not observe a significant imbalance in the relative indicators of cellular immunity in the early post-radiation period. Therefore, the use of only relative indicators of the immunogram cannot be recommended for assessing the IS state because they do not reflect the entirety of changes in cellular immunity. 

The expansion of knowledge about radio-induced effects on IS cells, as well as the study of the effects of irradiation regimens, the modern schemes of systemic chemotherapy, the stages of the oncological process, the somatic state of patients, and the initial state of the IS on the indicators of cellular immunity are important tasks in medical radiology.

The dynamic changes in cellular immunity and the composition of cell populations should undoubtedly be considered. Our results are of great clinical importance and should also be taken into account when assessing antitumor immunity due to the significant changes in T cell immunity identified.

Furthermore, the identified changes are of great importance if it is necessary to use specific targeted or immune therapy. It cannot be ruled out that a number of researchers single out RIL [13,14,15,16,17]. As a separate prognostically unfavorable factor for overall survival in certain groups of patients with BC, a continuous and more detailed approach to the study of immunity in cancer patients at different time points after RT is required.

Further studies are needed to determine the time periods and modes of RT suitable for BC and to assess the effects of various factors on the degree of development of RIL and the changes in the composition of lymphocyte populations in patients with BC.

## Figures and Tables

**Table 1:** The main parameters of the immunogram and their reference values.

Index	Reference Values
Lymphocytes (absolute count), ×10^9^/L	1.2–3.0
T-lymphocytes (CD3+), ×10^6^/mL	950–1800
T-lymphocytes (CD3+), %	55–80
T-helper cells (CD4+ CD3+), ×10^6^/L	570–1100
T-helper cells (CD4+ CD3+), %	31–51
Cytotoxic T cells (CD8+ CD3+), ×10^6^/L	450–850
Cytotoxic T cells (CD8+ CD3+), %	12–30
Immunoregulatory index (CD4+/CD8+), %	1.0–2.5
Natural killer cells (CD16+ CD56+ CD3−), ×10^6^/L	180–420
Natural killer cells (CD16+ CD56+ CD3−), %	4.0–18.0
B-lymphocytes (CD19+), ×10^6^/L	150–400
B-lymphocytes (CD19+), %	5.0–19.0

**Table 2:** Levels of lymphocytes in the studied groups of patients with breast cancer.

Level of Lymphocytes in Peripheral Blood	Without PCT (n = 33)		With PCT (n = 63)	
	Before EBRT (%)	After EBRT (%)	Before EBRT (%)	After EBRT (%)
<1.2 × 10^9^/L	22.2	75.0	14.0	77.0
1.2–3.0 × 10^9^/L	75.0	25.0	82.0	23.0
>3.0 × 10^9^/L	2.8	0	4.0	0

## Data Availability

The datasets generated and/or analyzed during the current study are available from Vasiliy I. Pustovoyt (vipust@yandex.ru) on reasonable request.
